# Evaluation of the clinical efficacy, safety, and permeability in pulmonary epithelial lining fluid of contezolid, a novel oxazolidinone drug, in adult patients with pneumonia

**DOI:** 10.1128/aac.01033-25

**Published:** 2026-03-04

**Authors:** Hailan Wu, Zixuan Cheng, Fengming Ding, Jianlan Hua, Hailin Wang, Xiannan Meng, Yanmei Cao, Yuancheng Chen, Yan Chen, Beining Guo, Tao Chen, Jing Zhang, Jing Zhang

**Affiliations:** 1 Institute of Antibiotics, Huashan Hospital, Fudan University 198171, Shanghai, People's Republic of China; 2 Key Laboratory of Clinical Pharmacology of Antibiotics, National Health Commission of the People’s Republic of China, Shanghai, People's Republic of China; 3 National Clinical Research Center for Geriatric Diseases, Huashan Hospital, Fudan University 159397, Shanghai, People's Republic of China; 4 Joint Laboratory of Hospital & Enterprise for Pathogen Diagnosis of Drug-resistant Bacterial Infections and lnnovative Drug R&D, Shanghai, People's Republic of China; 5 Department of Pulmonary and Critical Care Medicine, Zhongshan Hospital, Shanghai Medical College, Fudan University 12478, Shanghai, People's Republic of China; 6 Department of Respiratory and Critical Care Medicine, Shanghai General Hospital, Shanghai Jiao Tong University School of Medicine 12482, Shanghai, People's Republic of China; 7 Shanghai MicuRx Pharmaceutical Co. Ltd, Shanghai, People's Republic of China; 8 Clinical Pharmacology Research Center, Huashan Hospital, Fudan University 12478, Shanghai, People's Republic of China; 9 Shanghai Institute of Infectious Disease and Biosecurity, Fudan University 12478, Shanghai, People's Republic of China; Providence Portland Medical Center, Portland, Oregon, USA

**Keywords:** contezolid, pneumonia, pulmonary epithelial lining fluid, efficacy, safety, pharmacokinetics, penetration

## Abstract

Contezolid is a novel oxazolidinone antibiotic for the treatment of gram-positive bacteria, which are one of the most common pathogens of pneumonia. We conducted a prospective, single-center, open-label study to evaluate the clinical and microbiological efficacy, safety profile, and pulmonary epithelial lining fluid (ELF) penetration characteristics of contezolid in adult pneumonia patients. Sparse blood samples and bronchoalveolar lavage fluid samples were collected from patients after multiple oral doses of 800 mg of contezolid twice a day. Pharmacokinetic parameters were calculated by developing population pharmacokinetic (PopPK) modeling, and probability of target attainment was evaluated by Monte Carlo simulations. The study enrolled 15 patients (mean age 55 years) with primarily community-acquired pneumonia. Contezolid achieved a clinical cure rate of 80.0% and a bacterial clearance rate of 71.4%. Oral contezolid was well tolerated, and no drug-related adverse effects were observed in any of the subjects. The mean area under the concentration-time curve (AUC_₀–₁₂,ss_) was estimated by the PopPK model to be 33.06 mg·h/L in ELF and 71.95 mg·h/L in plasma. Assuming a plasma protein binding rate of 90% based on literature data, the ELF-to-free plasma AUC_0–12,ss_ ratio was 4.50. When the minimum inhibitory concentration was ≤4 mg/L, 800 mg of contezolid q12h could achieve the optimal therapeutic target in the plasma of patients with pneumonia. This study demonstrates that contezolid achieved excellent pulmonary penetration in adult patients with pneumonia.

## INTRODUCTION

Pneumonia represents a prevalent lower respiratory tract infection, broadly categorized as community-acquired pneumonia (CAP) or hospital-acquired pneumonia (HAP), the latter including ventilator-associated pneumonia (VAP). As the leading cause of hospitalization and mortality worldwide, CAP demonstrates an incidence of 1.5–14 cases per 1,000 population, while HAP occurs in 5–20 cases per 1,000 population (1, 2). Gram-positive pathogens, particularly *Streptococcus pneumoniae* and methicillin-resistant *Staphylococcus aureus* (MRSA), dominate the etiological spectrum (3, 4).

Linezolid, an oxazolidinone-class antibiotic, demonstrates efficacy against gram-positive pathogens, including MRSA and *S. pneumoniae*, with approved indications for both HAP and CAP. However, its clinical utility is limited by dose-dependent myelosuppression during extended therapy (≥14 days) (5). Contezolid, a novel oxazolidinone approved in China (2021), exhibits enhanced activity against resistant gram-positive strains (MRSA, methicillin-resistant *Staphylococcus epidermidis*, penicillin-resistant *S. pneumoniae*, and vancomycin-resistant *Enterococcus*) with improved safety profiles regarding hematological and neurological toxicity (6–8).

Pharmacokinetic (PK) evaluation of oral contezolid in healthy Chinese subjects showed a rapid peak plasma concentration at ~2 h, steady-state achievement by day 3 (q12h dosing), primary elimination via hepatic metabolism, and negligible renal excretion (0.8%–2.3% unchanged), with a terminal half-life of 2.08–4.84 h (9). Contezolid’s bioavailability is significantly influenced by food intake (9, 10). Fasting yields only 46.7%–60.6% of the fed-state bioavailability, whereas an 800 mg dose with a meal achieves 92.4%–100.0% relative bioavailability, along with a prolonged absorption lag time (22.5 min fasting to 47.5 min fed) and a shortened absorption half-life (69.4 min fasting to 59.3 min fed) (10). Dosage adjustments are unnecessary for elderly patients or those with renal impairment/mild-to-moderate hepatic dysfunction (11, 12). While currently approved only for complicated skin/soft tissue infections, its potential pulmonary applications require clinical evaluation.

For lower respiratory infections, antimicrobial efficacy critically depends on pulmonary penetration (13). Epithelial lining fluid (ELF) concentration serves as a key surrogate for assessing antibiotic distribution to infection sites (14). This study aims to evaluate the clinical efficacy and safety of contezolid in adult pneumonia patients with confirmed or suspected gram-positive infections as well as characterize steady-state ELF concentrations and pharmacokinetics following multiple dosing. The findings will provide crucial evidence for the potential role of contezolid in pneumonia management, addressing current therapeutic gaps in gram-positive pulmonary infections.

## RESULTS

**Fig 1:**
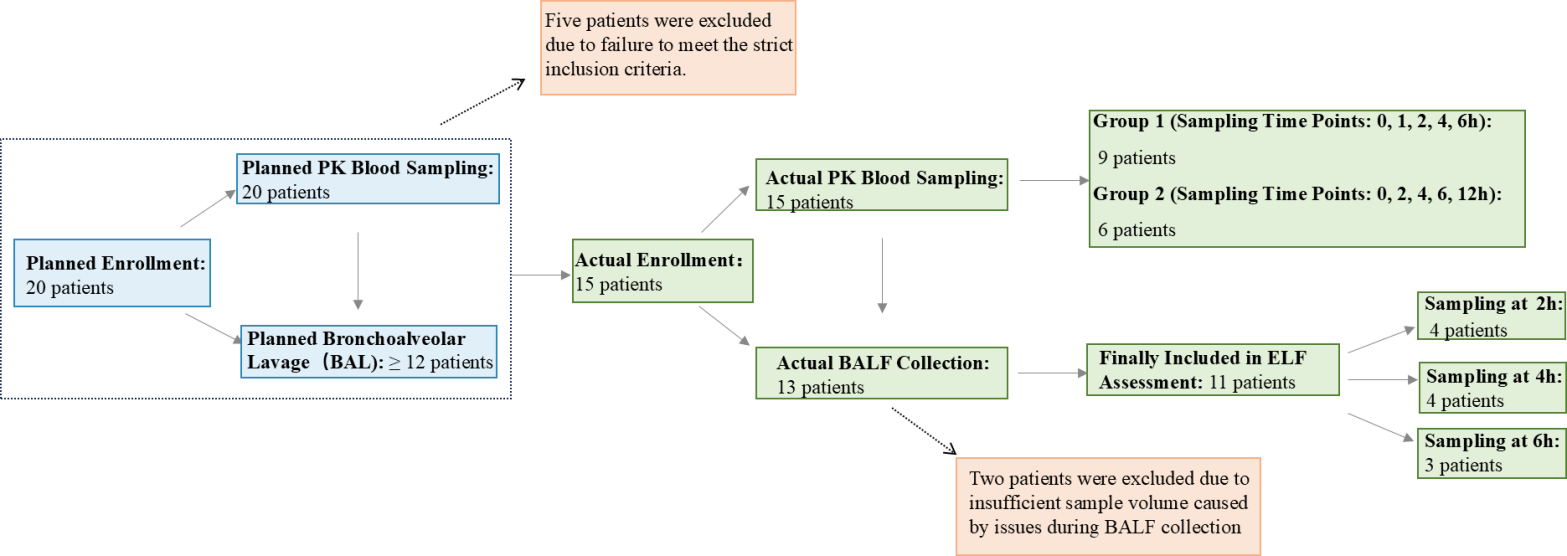
Patient enrollment and sample collection flow.

A total of 15 adult patients with clinically confirmed or suspected gram-positive bacterial pneumonia (nine male, six female) were ultimately enrolled and completed PK blood sampling in this study (Fig. 1). All enrolled patients received the study medication and were included in clinical efficacy, safety, and PK analyses. Bronchoalveolar lavage (BAL) was performed in 13 patients who had been under fasting conditions during drug administration, while the remaining two non-BAL patients received post-prandial medication. Two patients from the BAL group were excluded from ELF concentration analysis due to insufficient bronchoalveolar lavage fluid (BALF) volume resulting from collection issues (e.g., poor wedging or viscous secretions), leaving 11 patients included in the ELF permeability assessment. Patient enrollment and sample collection are shown in Fig. 1. Baseline demographic characteristics are presented in Table 1. Contezolid was well tolerated with no drug-related adverse events observed. Among the 15 pneumonia cases, the majority were CAP (*n* = 14), with only 1 case of HAP. Microbiological confirmation was achieved in seven patients, with the diagnosis in the remaining eight relying on clinical presentation and imaging. The predominant pathogen was *Staphylococcus aureus* (*n* = 3), followed by *Enterococcus faecalis* (*n* = 2), *Streptococcus pneumoniae* (*n* = 1), and *Streptococcus* spp. (*n* = 1). Furthermore, one patient had polymicrobial infections, including co-infections with gram-negative bacteria, such as *Klebsiella pneumoniae*. After 7–14 days of contezolid treatment, the clinical response rate was 80.0% (12/15), and the bacterial eradication rate was 71.4% (5/7) (Table 2).

**TABLE 1:** Demographic characteristics of the patients^*a*^

Characteristics	Value
Race	Asian
Age (years; mean, median [range])	55, 58 (36–73)
Gender (*n*, male/female)	9/6
Weight (kg; mean, median [range])	69, 65 (47–105)
Height (m; mean, median [range])	1.71, 1.67 (1.52–1.84)
Body mass index (kg/m^2^; mean, median [range])	24.5, 25.4 (17.3–29.3)
eGFR (mL/min; mean, median [range])	97.3, 96.0 (79–117)

^*a*^ eGRF, estimated glomerular filtration rate.

**TABLE 2:** Patient study completion and outcome^*a*^*^,^*^*d*^

Patient no.	HAP or CAP	Pathogens^*b*^	Treatment duration (day)	BAL sampling/day post-dose	Microbiological efficacy^*c*^	Clinical efficacy
1	CAP	\	14	No	\	Cure
2	CAP	\	7	Yes/4	\	Cure
3	CAP	*Staphylococcus aureus*	14	Yes/3	Eradication	Cure
4	CAP	*Streptococcus pneumoniae*	7	Yes/4	Eradication	Cure
5	CAP	*Staphylococcus aureus*	7	Yes/4	Eradication	Cure
6	CAP	*Staphylococcus aureus*	7	Yes/4	Persistence	Cure
7	CAP	\	14	Yes/3	\	Cure
8	CAP	\	14	Yes/3	\	Cure
9	CAP	\	7	Yes/3	\	Cure
10	CAP	\	7	Yes/4	\	Failure
11	CAP	\	7	Yes/4	\	Failure
12	CAP	*Enterococcus faecalis*	7	No	Eradication	Cure
13	CAP	*Streptococcus*	10	Yes/4	Presumed eradication	Cure
14	HAP	*Enterococcus faecalis*	7	Yes/3	Presumed persistence	Failure
15	CAP	\	7	Yes/4	\	Cure

^*a*^ CAP, community-acquired pneumonia; HAP, hospital-acquired pneumonia.

^*b*^ \, no bacteria detected.

^*c*^ \, not evaluated.

^*d*^ Patient 4 presented with* Klebsiella pneumoniae*.

**Fig 2:**
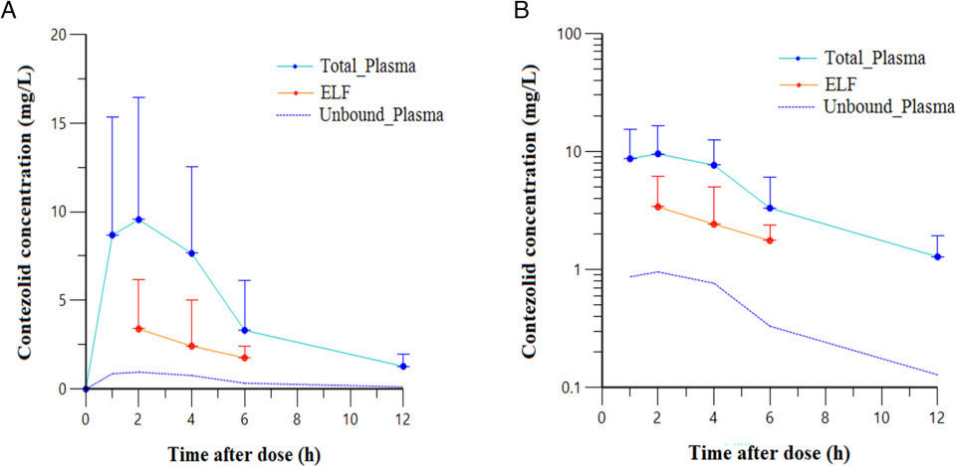
Mean (standard deviation) contezolid concentrations in plasma (total and unbound) and epithelial lining fluid (ELF) at the sample collection time points after multiple oral doses of 800 mg contezolid tablets in patients.(**A**) Linear scale graph. (**B**) Semilogarithmic scale graph. (Note : the unbound plasma concentration was calculated based on literature data of 90% plasma protein binding (11); fasting-state administration was required for bronchoscopy procedures).

Figure 2 displays the mean plasma concentration-time profile following multiple 800 mg oral doses of contezolid. The peak plasma concentration (*C*_max_) was observed in the sampling schedule that occurred at 2 h post-dose. Steady-state PK parameters calculated using non-compartmental analysis are shown in Table 3, revealing a mean *C*_max_ of 9.55 mg/L and a mean AUC_0–12,ss_ of 56.06 mg·h/L.

**TABLE 3:** PK parameters estimated from non-compartmental analysis in total plasma^*a*^

PK parameters	Unit	Mean
*C*_max_	mg/L	9.55
*C*_min_	mg/L	1.28
AUC_0–12,ss_	mg·h/L	56.06
*T*_max_	h	2
*T*_1/2_	h	3.33
*V*_ss_/*F*	L	68.52
CL_ss_/*F*	L/h	14.27

^*a*^ AUC_0–12,ss_, the area under the drug concentration-time curve from time 0 to 12 h at steady state; *C*_max_, peak plasma drug concentration; CL_ss_/*F*, apparent clearance at steady state; *T*_max_, time to reach peak plasma concentration; *T*_1/2_, half-life; *V*_ss_/*F*, apparent volume of distribution at steady state.

**Fig 3:**
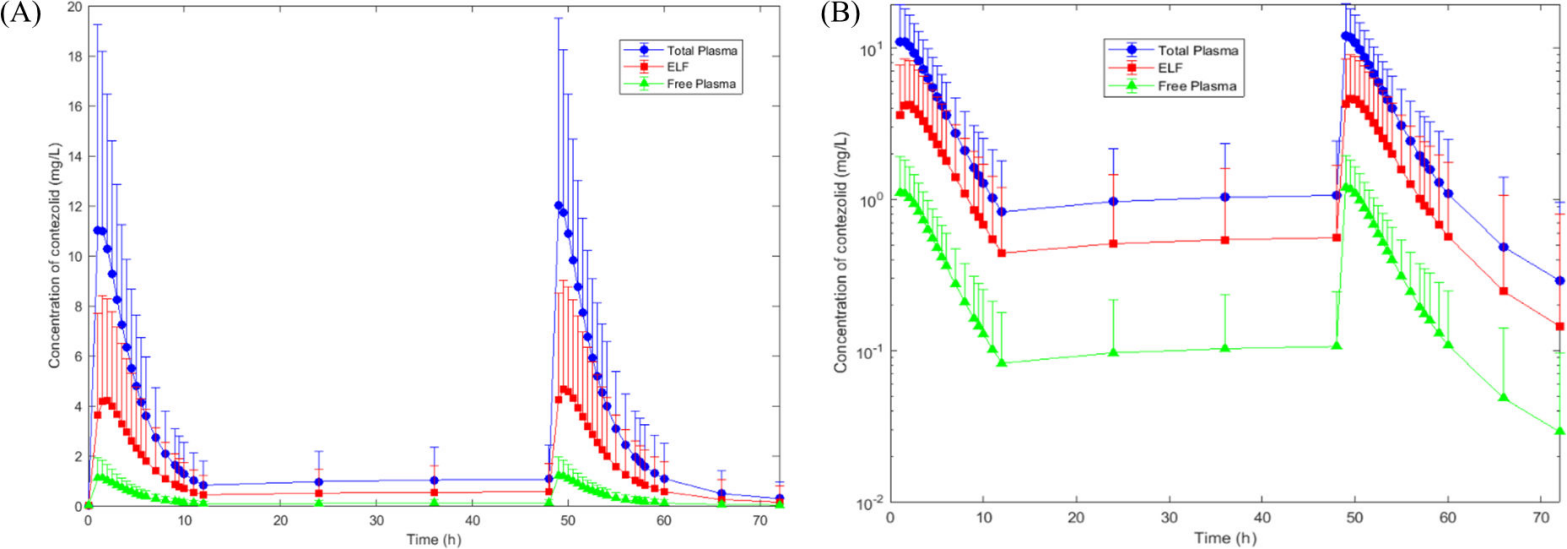
The concentration-time curves of contezolid in plasma and ELF after multiple oral doses of 800 mg contezolid tablets in patients with pneumonia. Values are expressed as the mean ± standard deviation. (**A**) Linear scale graph. (**B**) Semilogarithmic scale graph.

A total of 75 measurable blood drug concentrations from all 15 subjects and 11 ELF concentrations from a subset of 11 subjects were used to construct the population PopPK model for contezolid. The covariate formula is presented in the supplemental file. Fasting was the covariate of bioavailability and albumin for the absorption rate of contezolid. The parameters and bootstrapping of contezolid derived from the final PopPK model are provided in Table S1. The goodness-of-fit diagram and visual predictive check (VPC) are shown in Fig. S2 and S3, respectively. The concentration-time curves in total plasma and ELF were simulated using the final PopPK model, and the total plasma concentrations were subsequently corrected for 90% protein binding (11) to yield the free drug concentrations. As shown in Fig. 3, the concentration-time curve of ELF followed similar temporal trends to plasma. During the sampling period within the dosing interval of 12 h, ELF concentrations exceeded free plasma concentrations but remained below total plasma concentrations. Table 4 showed that the ELF-to-total plasma concentration ratio ranged from 0.35 to 0.71, while the ELF-to-unbound plasma concentration ratio ranged from 3.47 to 7.12. Based on AUC_0–12,ss_, the mean penetration ratios were 0.45 (total drug) and 4.50 (free drug), respectively.

**TABLE 4:** Concentrations and AUC_0–12,ss_ of contezolid in ELF comparing to plasma levels^*a*^*^,^*^*b*^

Time (h)	Concentration (mg/L; mean ± SD [median])			ELF to plasma ratio (mean ± SD [median])	
	Total plasma	Unbound plasma	ELF	ELF to total plasma	ELF to unbound plasma
2	*n* = 4	*n* = 4	*n* = 4	*n* = 4	*n* = 4
	6.15 ± 4.41 (6.70)	0.62 ± 0.44 (0.67)	3.39 ± 2.76 (3.32)	0.50 ± 0.13 (0.50)	5.02 ± 1.28 (4.98)
4	*n* = 4	*n* = 4	*n* = 4	*n* = 4	*n* = 4
	6.31 ± 1.06 (6.00)	0.63 ± 0.11 (0.60）	2.42 ± 2.59 (1.49)	0.35 ± 0.31 (0.26)	3.47 ± 3.11 (2.56)
6	*n* = 3	*n* = 3	*n* = 3	*n* = 3	*n* = 3
	5.51 ± 3.67 (6.60)	0.55 ± 0.37 (0.66)	1.76 ± 0.629 (1.48)	0.71 ± 0.91 (0.22)	7.12 ± 9.07 (2.23)
AUC_0–12,ss_(h·mg/L)	71.95 ± 36.56 (61.05)	7.20 ± 3.66 (6.11)	33.0 ± 43.58 (22.42)	0.45 ± 0.36 (0.35)	4.50 ± 3.58 (3.52)

^*a*^ AUC_0–12,ss_, the area under the drug concentration-time curve from time 0 to 12 h at steady state; ELF, epithelial lining fluid; SD, standard deviation.

^*b*^ Unbound plasma concentrations were calculated assuming a plasma protein binding rate of 90%, as reported in the literature (11).

**Fig 4:**
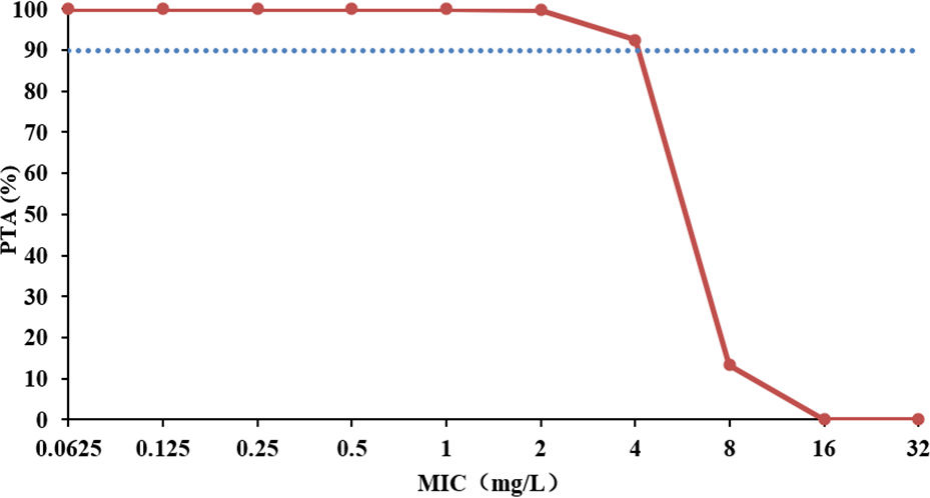
Probability of target attainment for contezolid after multiple oral doses of 800 mg contezolid tablets in patients with pneumonia. The blue dotted line indicates 90% probability of target attainment. MIC, minimum inhibitory concentration; PTA, probability of target attainment.

The probability of target attainment (PTA) for contezolid after multiple oral doses of 800 mg contezolid tablets in patients with pneumonia using Monte Carlo simulations is shown in Fig. 4. The result showed that increasing the minimum inhibitory concentration (MIC) was associated with a reduced PTA. In the simulations for MIC ≤4 mg/L in patients with pneumonia, an *f*AUC_0–24_/MIC ≥2.3 (15) was achieved in ≥90% of the patients with 800 mg q12h.

## DISCUSSION

Linezolid, a representative oxazolidinone antibiotic, has demonstrated efficacy against both methicillin-sensitive *Staphylococcus aureus* and MRSA and *Streptococcus pneumoniae* pneumonia, with well-characterized PK of linezolid in pneumonia patients and ELF penetration in the human body (16–21). Currently, there has been no research report on the relevant situation of the new oxazolidinone drug contezolid in patients with pulmonary infections. As the first study evaluating the novel oxazolidinone contezolid for pulmonary infections, we systematically assessed its clinical efficacy, safety profile, and pulmonary penetration in adult pneumonia patients. *S. aureus* constituted about 50% of baseline gram-positive isolates, with contezolid achieving bacterial eradication and clinical cure rates of 66.7% and 80.0%, respectively, demonstrating favorable therapeutic outcomes for adult pneumonia. Notably, no drug-related adverse events were observed, indicating excellent tolerability.

In this study, the non-compartment model was used to analyze the pharmacokinetic characteristics of contezolid in pneumonia patients. It should be noted that the observed *T*_max_ of 2 h for both plasma and ELF should be interpreted with caution due to the limited number of sampling time points around the anticipated peak concentration. The results revealed reduced contezolid exposure in pneumonia patients compared to both skin and soft tissue infection patients (22) (*C*_max_ decreased by 34.6% [9.55 vs 14.61 mg/L]; AUC_0–12,ss_ decreased by 28.5% [56.06 vs 78.37 mg·h/L]) and healthy volunteers (9) (*C*_max_ decreased by 63.9% [9.55 vs 26.45 mg/L]; AUC_0–12,ss_ decreased by 38.0% [56.06 vs 90.38 mg·h/L]). This PK alteration primarily stems from fasting-state administration (required for bronchoscopy procedures) and disease-related gastrointestinal dysmotility. The bioavailability of contezolid in the fasting state decreased by 54.6% compared with that in the state of a high-fat meal (9, 22). Among the 15 pneumonia patients in this study, 13 patients underwent bronchoalveolar lavage. They took the medicine on an empty stomach after fasting before the operation, and then samples were collected. However, both the healthy subjects and patients with skin and soft tissue infections in the literature took the medicine after meals, and the healthy subjects had better gastric absorption and higher drug exposure. Despite reduced plasma exposure, the mean *f*AUC_24h_/MIC_90_ ratio (2 × *f*AUC_0–12,ss_ / 2 = 7.20) in plasma remained above the target value of 2.3 (15) for optimal efficacy against *S. aureus* (MIC_90_ = 2 mg/L) (23). When MIC ≤4 mg/L, 800 mg of contezolid q12h could achieve the optimal therapeutic target in plasma of patients with pneumonia.

Since the bronchoalveolar lavage fluid was collected within the first 6 h after drug administration for patients undergoing bronchoalveolar lavage, it was necessary to establish a PopPK model of contezolid in plasma and pulmonary ELF to calculate the AUC_0–12,ss_ in the ELF. During the structural model development, a physiologically based three-compartment model comprising central, ELF, and peripheral compartments was initially evaluated. However, parameter estimation indicated a negligible volume for the peripheral compartment (*V*_p_ = 0.1 L), and its inclusion did not significantly reduce the objective function value (ΔOFV = 2.3, *P* > 0.05). Consequently, the peripheral compartment was excluded, resulting in a final two-compartment structure consisting of central and ELF compartments. Additionally, incorporation of a free drug fraction parameter for drug transfer between central and ELF compartments was tested; the estimated fraction was 1, suggesting no substantial impact on model fit, and this parameter was therefore omitted. Inclusion of study site as a random effect significantly enhanced model stability and reduced the OFV from –133 to –138. Covariate analysis identified a significant effect of food status on bioavailability, consistent with previous literature (9, 10, 22). Furthermore, baseline serum albumin level showed a positive correlation with the absorption rate constant.

The mean penetration ratios of contezolid in ELF relative to total plasma concentrations were 0.50, 0.35, and 0.71 at 2, 4, and 6 h post-dose, respectively. The steady-state area under the concentration-time curve from 0 to 12 h (AUC_₀–₁₂,ss_) showed a mean penetration ratio of 0.45. Since only unbound drug molecules are capable of extravasation into the pulmonary ELF, protein binding-adjusted penetration was evaluated. After correction for plasma protein binding (contezolid 90% [11]), the ELF-to-free plasma concentration ratios ranged from 3.47 to 7.12 across sampling points, with an AUC-based penetration ratio of 4.50, indicating efficient lung targeting and a 4.50-fold higher free drug exposure in ELF compared to plasma. In comparison, published data on linezolid, a representative oxazolidinone, show variable lung penetration. In healthy volunteers, linezolid achieved an ELF-to-plasma ratio of 2.3–4.2 after oral administration (16). However, lower ratios were reported in critically ill patients receiving intravenous therapy: approximately 1.04 in ventilator-associated pneumonia (17), a median of 0.97 under continuous infusion (18), 1.7 in pneumonia-associated sepsis (19), and medians of 0.80 (intermittent) and 1.06 (continuous) in obese VAP patients (20). Notably, Kiem and Schentag (21) suggested that conventional bronchoalveolar lavage methods may overestimate linezolid penetration, with uncorrected ratios ranging from 1.5 to 12.1; after technical correction, the ELF-to-free plasma ratio stabilized around 2.0 (21). The observed difference in pulmonary distribution between contezolid and linezolid may be largely attributed to their distinct plasma protein binding (90% vs 31%, respectively), in addition to influences from the study population, dosing regimen, and critical illness. From a pharmacodynamic perspective, contezolid concentrations in ELF consistently exceeded the corresponding free plasma levels throughout the dosing interval. Moreover, ELF concentrations remained above the MIC_90_ (1–2 mg/L) for gram-positive bacteria for over 6 h, supporting adequate lung exposure and potential bactericidal activity at the infection site. These findings provide a robust pharmacokinetic rationale for the efficacy of contezolid in pulmonary infections.

In this study, due to the need for patients with pneumonia to take medicine on an empty stomach before undergoing bronchoscopy, the bioavailability of the drug was low, and the concentration of contezolid in ELF was also relatively low. Under routine clinical practice, pneumonia patients who are not affected by surgery can take medicine after meals to increase the bioavailability of contezolid and improve its concentration in ELF. Looking ahead, the development of intravenous formulations represents a promising strategy to overcome these absorption limitations and expand the applicable population, such as patients with severe pneumonia.

This study has several limitations that warrant consideration. First, the relatively small cohort size and limited number of samples may restrict the generalizability of our findings. Second, the PK sampling strategy employed sparse time-point collection from different subjects rather than intensive blood sampling from each individual, necessitating the construction of composite concentration-time curves through data pooling. Consequently, the PK parameters in this study failed to calculate the coefficient of variation and the standard deviation. Third, due to the lack of established PK/PD targets for the lung/ELF compartment, no target-based PK/PD analysis for ELF was performed.

### Conclusion

This study provides preliminary data indicating that oral contezolid exhibits relatively good efficacy, safety, and favorable intrapulmonary pharmacokinetics as evidenced by good pulmonary ELF penetration in the treatment of adult pneumonia in a limited number of patients. These results warrant further clinical investigation of contezolid for gram-positive bacterial pulmonary infections, including MRSA-associated pneumonia.

## MATERIALS AND METHODS

### Study design and research subjects

Hospitalized patients aged ≥18 years, either male or female, who were clinically diagnosed with HAP or CAP and required contezolid tablets for the treatment of pneumonia, were included. Microbiological tests were required at the time of baseline admission. Key exclusion criteria included viral or atypical pneumonias (pneumocystis or tuberculosis); structural lung diseases (e.g., bronchiectasis and cystic fibrosis); monomicrobial gram-negative infections (mixed infections with gram-positive predominance were permitted with adjunctive gram-negative coverage); bloodstream infections; critical illness requiring mechanical ventilation or vasopressors; severe CAP (PORT/PSI class V) requiring ICU admission; recent antibiotic use (>48 h within 72 h); oxazolidinone hypersensitivity; or human immunodeficiency virus positivity.

The study planned to enroll 20 eligible patients from Zhongshan Hospital Affiliated to Fudan University and Shanghai First People’s Hospital, with all patients receiving oral contezolid 800 mg every 12 h for 7–14 days. During the initial 72-h period, aztreonam therapy was permitted as clinically indicated for gram-negative coverage. Blood samples for PK analysis were collected from all participants. A subset (≥12 patients) underwent BAL post-dose day 5 or 6 to assess pulmonary ELF penetration.

Patients were required to take the medicine after meals, except when fasting was required prior to BAL procedures.

### Sample collection

The enrolled patients were randomly allocated into two groups for pharmacokinetic sampling. Baseline blood samples were collected from all participants prior to the initial dose administration. Following administration of the fifth or sixth dose, serial blood samples were obtained at specified intervals: group 1 at 1.0 ± 0.17, 2.0 ± 0.17, 4.0 ± 0.17, and 6.0 ± 0.17 h post-dose; group 2 at 2.0 ± 0.17, 4.0 ± 0.17, 6.0 ± 0.17, and 12.0 ± 0.17 h post-dose. All blood samples were collected in EDTA-containing tubes, immediately processed by centrifugation, and the harvested plasma aliquots were stored at −70°C until analysis.

For pulmonary penetration assessment, the eligible patients were stratified into three cohorts (*n* ≥ 4 per group) for BAL procedures at 2.0 ± 0.17, 4.0 ± 0.17, or 6.0 ± 0.17 h post-dose (fifth or sixth administration). Before bronchoscopy, patients were fasted for ≥8 h, lying flat on the operating bed, and peripheral venous access was established. After induction of anesthesia, a laryngeal mask was placed through the mouth, fixed with dental pad, and connected to the anesthesia machine for mechanical ventilation, and heart rate, blood pressure, and pulse oximetry (SpO_2_) were continuously monitored. A fiberoptic bronchoscope was inserted through the laryngeal mask into the bronchial opening of the middle lobe of the right lung, and BAL was performed four times: 50 mL of sterile saline at 37°C was injected through the biopsy channel each time and then sucked back into a sterile container at a negative pressure of ≤20 mmHg. The first recovered fluid (50 mL) was discarded to minimize contamination, and the rest of the lavage fluid was combined into BALF, with a total recovery rate of ≥30%, and the specimen was stored on ice throughout the whole process. Concurrently with the BAL procedure, paired blood samples were collected from the patient’s peripheral vein into serum separation tubes, left undisturbed for 30 min at room temperature, and centrifuged (1,600 × *g*, 10 min) to isolate the serum. All BALF and serum samples are aliquoted, frozen at −80°C within 1 h, and reserved for subsequent detection. The urea correction method was employed to calculate ELF concentrations using the formula CTZ_ELF_ = CTZ_BALF_ × Urea_serum_ / Urea_BALF_, where CTZ_ELF_ represents the antibiotic concentration in ELF; CTZ_BALF_ is the measured drug concentration in BALF; Urea_serum_ is the serum urea concentration; and Urea_BALF_ is the urea concentration in BALF.

### Determination of the concentration of contezolid in plasma, serum, and BALF

A validated ultra-performance liquid chromatography-tandem mass spectrometry (UPLC-MS/MS) method was utilized for quantification of contezolid in plasma and BALF. The analytical system consisted of an ACQUITY UPLC chromatograph (Waters Corporation, USA) coupled with an API 4000 triple quadrupole mass spectrometer (AB Sciex, USA) equipped with an electrospray ionization source. Chromatographic and mass spectrometric conditions were established based on our previously published methodology (24). Quantification was performed using multiple reaction monitoring of the following transitions: *m*/*z* 409.2→269.1 for contezolid and *m*/*z* 414.1→146.1 for the internal standard (D_5_-contezolid).

For plasma analysis, the method demonstrated linearity over the concentration range of 0.0100–5.00 mg/L, with a lower limit of quantification of 0.0100 mg/L. Sample preparation involved liquid-liquid extraction as previously described (24).

BALF analysis showed identical linearity (0.0100–5.00 mg/L) with quality control samples prepared at three concentrations (0.0300, 0.400, and 4.00 mg/L). The method exhibited satisfactory precision (1.6%–3.7%) and accuracy (97.0%–107.5%) across the calibration range. BALF samples were processed as follows: 20 μL aliquots were mixed with 280 μL of internal standard solution (0.100 mg/L D_5_-contezolid in acetonitrile), vortexed, and centrifuged at 4,500 rpm for 10 min. Subsequently, 200 μL of supernatant was transferred to a 96-well plate, evaporated to dryness under nitrogen at 40°C, and reconstituted in 300 μL of 30% acetonitrile-water solution. After vortex mixing, 5 μL was injected for liquid chromatography-tandem mass spectrometry analysis.

### Determination of the concentrations of urea in serum and BALF

Urea concentrations in serum and BALF were determined using a validated UPLC-MS/MS method developed in our laboratory (25). Serum urea analysis employed a [^15^N₂]-urea calibration curve (50.0–1,500.0 mg/L), while BALF measurements used a lower range (2.00–100.0 mg/L), with [^13^C, ^15^N₂]-urea as internal standard. The method incorporated a correction factor derived from the response ratio of ^15^N₂-urea to natural urea at 1 mg/L to ensure accurate quantification in both matrices.

### Pharmacokinetic analysis

Plasma pharmacokinetic parameters of contezolid at steady state were derived using non-compartmental analysis with Phoenix WinNonlin software (v.8.1). Key parameters included maximum and minimum plasma concentrations (*C*_max_ and *C*_min_), area under the concentration-time curve from 0 to 12 h at steady state (AUC_0–12,ss_) and extrapolated to infinity (AUC_0–∞_), time to maximum concentration (*T*_max_), terminal half-life (*T*_1/2_), apparent volume of distribution (*V*_z_/*F*), and apparent systemic clearance (CL_ss/_*F*).

### Population PK analysis

The PopPK model was developed in both plasma and ELF using NONMEM 7 (v.7.5.6, ICON Development Solutions), employing the first-order conditional estimation with interaction method. The distribution and elimination of contezolid in plasma and pulmonary ELF were described by a two-compartment model (Fig. S1). Drug absorption and transport between central and ELF compartments were all consistent with first-order kinetics. Covariate screening was carried out by forward inclusion and backward elimination method. The final model was evaluated by goodness of fit and was validated by VPCs and bootstrap. The AUC_0–12h,ss_ was determined for contezolid in plasma and ELF by simulating. Each subject was simulated 200 times using NONMEM. A comprehensive description of the methodology, including the pharmacokinetic model structures and the associated differential equations, is provided in the supplemental file. The differential equations are as follows.

### PK/PD analysis

The steady-state AUC_0–24h,ss_ of contezolid was simulated following an 800 mg q12h regimen using the final PopPK model in NONMEM (v.7.5.6). The target value of the PK/PD index (*f*AUC_0–24h_/MIC) was set as 2.3 (15) against *S. aureus* (free fraction of contezolid used was 10% [11]). Monte Carlo simulations (*n* = 5,000) were employed to calculate the PTA based on various MICs (0.25–32.0 mg/L [23]). The PTA for achieving *f*AUC_0–24h_/MIC was calculated by Monte Carlo simulations for the steady-state plasma.

### Efficacy evaluation

The clinical evaluation protocol incorporated serial assessments of pneumonia symptoms, physical examination findings, laboratory parameters, and radiographic imaging performed according to the predetermined visit schedule. Primary clinical efficacy, determined at 7–14 days of post-treatment evaluation using stringent cure criteria, required (i) complete resolution or return to preinfection status of all baseline symptoms and signs, (ii) normalization of relevant laboratory markers, (iii) radiographic evidence of inflammatory infiltrate resolution or significant improvement, and (iv) clinical determination that further antimicrobial therapy was unnecessary. Cases were classified as treatment failures if they met any of the following criteria: persistence or worsening of baseline abnormalities, disease progression with new clinical or radiographic findings, development of purulent complications (empyema or lung abscess), or requirement for alternative antimicrobial agents.

Microbiological assessment employed a comprehensive diagnostic approach combining conventional culture methods (respiratory secretion and blood cultures) with advanced molecular techniques (pathogen capture metagenomics of deep sputum and bronchoalveolar lavage specimens). Bacteriological outcomes were categorized as confirmed eradication (baseline pathogen not detected post-treatment), presumed eradication (clinical cure with unavailable follow-up specimens), confirmed persistence (baseline pathogen identified post-treatment), or presumed persistence (clinical failure with unavailable cultures), with eradication rates calculated from both confirmed and presumed eradication cases.

### Safety evaluation

The safety analysis included all enrolled participants who received at least one dose of contezolid (safety population). A comprehensive safety assessment was conducted throughout the study period, incorporating serial physical examinations, vital sign monitoring, 12-lead electrocardiograms, and standardized laboratory tests. All adverse events were systematically documented and evaluated for severity (graded according to CTCAE v.5.0 criteria), duration, and potential relationship to study treatment (assessed using the WHO-UMC causality assessment system).

## Data Availability

The data supporting the findings of this study are available from the corresponding authors upon reasonable request.

## SUPPLEMENTAL MATERIAL

Supplemental material aac.01033-25-s0001.docx Fig. S1 to S3; Table S1.

ASM does not own the copyrights to Supplemental Material that may be linked to, or accessed through, an article. The authors have granted ASM a non-exclusive, world-wide license to publish the Supplemental Material files. Please contact the corresponding author directly for reuse.
