# Supplementary material for: Evaluation of the clinical efficacy, safety, and permeability in pulmonary epithelial lining fluid of contezolid, a novel oxazolidinone drug, in adult patients with pneumonia
Source: Antimicrob Agents Chemother. 2026 Mar 4;70(4):e01033-25. doi: 10.1128/aac.01033-25 (PMC13041416; doi:10.1128/aac.01033-25)
Supplement: Supplemental material — Fig. S1 to S3; Table S1. [file aac.01033-25-s0001.docx]

**Supplemental Materials**

**Study Title:** Evaluation of the clinical efficacy, safety and permeability in pulmonary epithelial lining fluid of contezolid, a novel oxazolidinone drug, in adult patients with pneumonia

**Supplementary Details on Population PK Analysis (MATERIALS AND METHODS):**

Each PK parameter conformed to a lognormal distribution :

Para_Ind_ =Para_TV_*exp(ETA)

where Para_TV_ and Para_Ind_ represent typical and individual values of PK parameters, respectively. ETA indicates interindividual variability (IIV) with mean zero and variance ω^2^ .

A site-stratified proportional error model was used for residual variability. The relationship between observations (Y) and individual predictions (F) was defined as:

Plasma: Y = F+F*ERR1*(1-site)+F*ERR2*site

ELF: Y = F+F*ERR3*(1-site)+F*ERR4*site

where site is an indicator variable (0 for Zhongshan Hospital, 1 for First Hospital). ERR1 to ERR4 are mutually independent proportional error terms, each with a mean of zero and distinct variances (σ²₁ to σ²₄).

Covariate screening was caried out by forward inclusion and backward elimination method. A covariate was considered statistically significant in forward inclusion when P < 0.05 (OFV decreased by 3.84) and in backward elimination when P < 0.001 (OFV increased by 10.83). The effects of the following covariates on the PK parameters of contezolid were tested: age, sex, height, weight, BMI, fasting, albumin, alkaline phosphatase, total bilirubin, serum creatinine, creatinine clearance, eGFR, alanine aminotransferase, aspartate aminotransferase, site.

The pharmacokinetic models and differential equations were as follows:

| 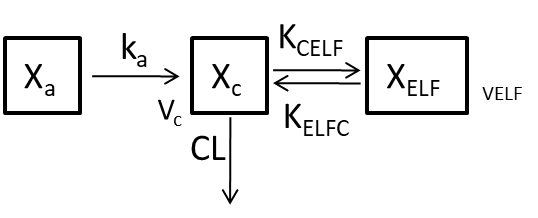 | If T≤TLAG,   If T>TLAG,    Initial condition:  X_a,0_=Dose*BIO  X_c,0_=0  X_ELF,0_=0 |
| --- | --- |

Figure1.Pharmacokinetic model (left) and differential equations (right) of contezolid in plasma and ELF. X_a_, X_c_ and X_ELF_ denote the amount of contezolid in absorption, central and peripheral and lung ELF compartment, respectively. K_a_ represents the absorption rate of contezolid. K_CELF_ is the transit rate constant from the central to lung ELF, K_CELF_ is the transit rate constant from the lung ELF to central. BIO, bioavailability. CL__CORR_ is the bioavailability-corrected apparent clearance, V_C_CORR_ is the bioavailability-corrected apparent central volume of distribution, V_ELF_CORR_ is the bioavailability-corrected apparent epithelial lining fluid volume of distribution, C_plasma_ is the concentration of contezolid in plasma, C_ELF_ is the concentration of contezolid in ELF.

**Supplementary Details on Population PK Analysis (RESULTS):**

The final PopPK model was shown as follows. ALB affected Ka, FASTING (value=0: fast, value=1: food) affected BIO.

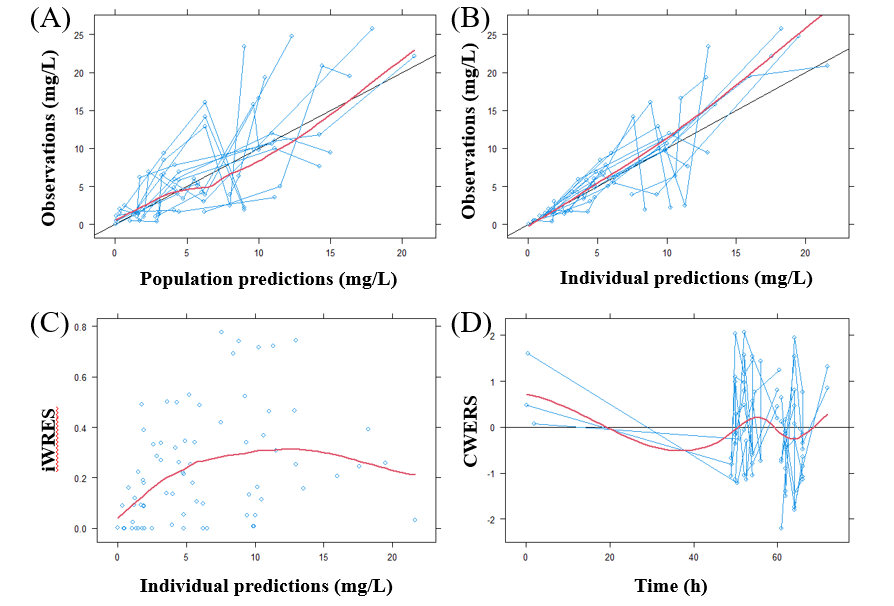


Figure 2. Goodness-of-fit plots of the final PopPK model of contezolid. iWRES: individual weighted residual. CWRES: Conditional weighted residuals. Blue circle: actual data. Black line: unity line (A, B) or zero horizontal line (D). Red line: locally weighted linear regression.


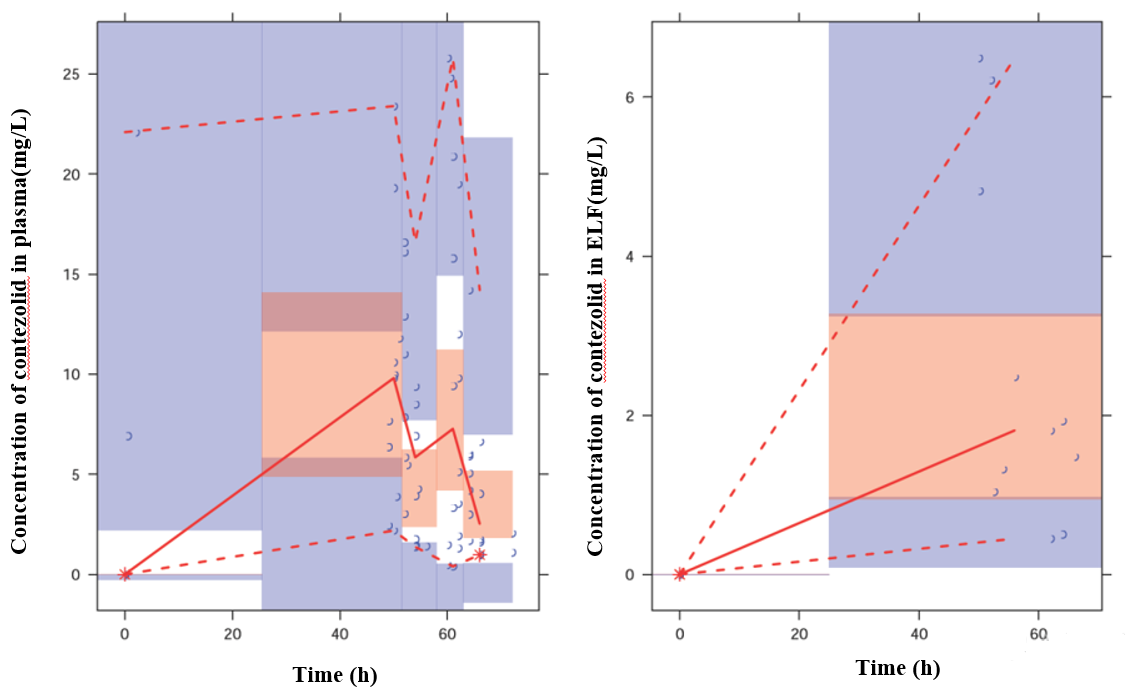


Figure 3. Visual predictive check of the final PopPK model of contezolid. Red solid line, median of observed data; red shaded area, 95% confidence interval of predicted concentrations; red dashed lines, 95% and 5% percentiles of observed data; blue shaded area, corresponding to 95% confidence interval.

| Table1. Parameter estimates and bootstrapping of the final population pharmacokinetic model of contezolid | | | | | |
| --- | --- | --- | --- | --- | --- |
| Parameter(unit) | Definition | Estimate based on original dataset | Estimate based on  bootstrap dataset | | Bias（%） |
|  |  | Mean(shrinkage%) | Mean | RSE（%） |  |
| K_a_ (1/h) | The absorption rate | 0.48 | 0.54 | 53.8 | 11.8 |
| CL (1/h) | The apparent clearance | 9.42 | 9.43 | 12.6 | 0.1 |
| V_c_ (L) | The apparent distribution volume in the central | 13.11 | 12.53 | 27.0 | -4.4 |
| K_CELF_ (1/h) | The transfer rate constant from the central to ELF | 0.99 | 1.00 | 2.5 | 0.8 |
| K_ELFC_ (1/h) | The transfer rate constant from the ELF to central | 2.99 | 2.98 | 4.7 | -0.2 |
| V_ELF_ (L) | The distribution volume in the ELF | 12.82 | 21.21 | 32.5 | -4.8 |
| T_LAG_ (h) | Lag time in absorption | 0.083 | 0.081 | 145.0 | 0.9 |
| BIO(FASTING) | Power exponent of FASTING on BIO | 1.83 | 1.90 | 19.6 | 3.9 |
| K_a_(ALB) | Power exponent of ALB on K_a_ | 8.02 | 6.89 | 39.3 | -14.1 |
| ω^2^ _Ka_ | Interindividual variability of K_a_ | 0.839 (14.8) | 0.72 | 76.4 | -13.7 |
| ω^2^ _KCELF_ | Interindividual variability of K_CELF_ | 0.47 (13.8) | 0.41 | 44.9 | -12.3 |
| π^2^ _BIO_ | Inter-occasion variability of BIO | 0.066 (46.0) | 0.054 | 83.7 | -18.8 |
| σ^2^ Zhongshan_Plasma | Zhongshan_Plasma proportional residual error | 0.26 (11.0) | 0.29 | 52.7 | 12.0 |
| σ^2^ First_hospital_Plasma | First_hospital_Plasma proportional residual error | 0.13 (13.8) | 0.13 | 69.4 | 1.5 |
| σ^2^ Zhongshan_ELF | Zhongshan_ELF proportional residual error | 1.00E-06 (99.7) | 6.17E-03 | 1901.1 | 6.17E+05 |

_Bias (%) = (Para_bootstrap_dataset/Para_original_dataset-1)×100%, Para means parameter estimate._
